# Isolation and characterization of a novel lytic bacteriophage for *Faecalibacterium prausnitzii,* a key member of the human gut microbiota

**DOI:** 10.1080/29933935.2026.2708416

**Published:** 2026-07-29

**Authors:** Mikaela Whitty, Maria Jelinic, Grant R. Drummond, Antony Vinh, Steve Petrovski

**Affiliations:** a Department of Microbiology, Anatomy, Physiology & Pharmacology, La Trobe University Bundoora, VIC, Australia; b La Trobe Institute for Molecular Science (LIMS), La Trobe University, Bundoora, VIC, Australia

**Keywords:** *Faecalibacterium prausnitzii*, bacteriophage, lytic phage, gut microbiota, host–phage interaction

## Abstract

*Faecalibacterium prausnitzii* is a dominant and health-associated member of the human gastrointestinal tract that is consistently depleted in individuals with metabolic, cardiovascular, and psychiatric disorders. Despite its importance in gut health, little is known about the bacteriophages (or phages) that infect this species. Phages are increasingly recognized as key regulators of bacterial population dynamics in the gut microbiota; however, while prophages of *F. prausnitzii* have been described, no lytic phages targeting this bacterium have been reported. Here, we describe the isolation and characterization of the first lytic *F. prausnitzii* bacteriophage, vB_Fpr_FP01. Whole-genome sequencing and comparative genomics analysis demonstrate that FP01 represents a novel species, clustering within a genus that includes an uncharacterized phage within the class Caudovirales. Comparative analysis also identified closely related viral sequences in the human gut virome dataset, suggesting that related phages occur in gut-associated environments. These findings expand the current knowledge of *F. prausnitzii*-associated phages and provide a framework for future studies investigating phage‒host interactions and their role in shaping gut microbial communities.

## Introduction

*Faecalibacterium prausnitzii* is a predominant commensal bacterium of the human gastrointestinal tract, typically comprising 5%–15% of the total bacterial community in the mammalian gut.^1^ This Gram-positive, mesophilic, rod-shaped, obligate anaerobic bacterium is considered a key contributor to intestinal health and is frequently cited as a biomarker of a healthy gut microbiota. Notably, a reduced *F. prausnitzii* abundance has been reported across a wide range of gastrointestinal, metabolic, cardiovascular, and psychiatric disorders.^2-11^ Despite its recognized importance, experimental investigation of *F. prausnitzii* remains challenging, largely because of its extreme oxygen sensitivity, which has limited cultivation and mechanistic studies.^12^ Consequently, it remains unclear whether the reduced population of *F. prausnitzii* is a cause or consequence of disease-associated physiological changes.

The gut microbiota plays a central role in host physiology, contributing to essential metabolic processes, including digestion, metabolite synthesis, and immune protection.^13-15^ The composition and diversity of this microbial community are shaped by numerous factors, such as diet, antibiotic use, host’s immune system, and geographical location, which in turn influence microbial spatial distribution and abundance within the gut.^16-19^ Perturbations to these tightly regulated microbial networks are increasingly associated with disease states, highlighting the need to understand regulators of key bacterial taxa within this ecosystem.

Among these regulators, bacteriophages (or phages) are now recognized as major drivers of bacterial population dynamics in the gut microbiota. The gastrointestinal tract contains approximately 10^13^ viral particles, with an estimated 97.7% representing phages.^20^ ^,^ ^21^ Phages are obligate bacterial parasites and can adopt either lytic or lysogenic lifestyles. Lytic phages replicate within and ultimately lyse their bacterial hosts, directly shaping bacterial abundance through predation. In contrast, lysogenic phages integrate into the host genome as a prophage, where they can influence bacterial gene expression, enhance host fitness, and can passively replicate within the bacterial chromosome.^22^ Under specific stress conditions, prophages may be induced to enter the lytic cycle, linking environmental cues to bacterial population turnover.^23-25^ Through these mechanisms, both lytic and lysogenic phages contribute to structuring microbial communities within the gut.

Although the contribution of phages to gut health and disease remains underexplored, emerging evidence suggests that changes in phage community dynamics are associated with pathological states. Healthy individuals typically harbor a relatively stable gut virome, often characterized by abundant bacteriophage populations such as CrAss-like phages and members of the *Microviridae*. However, gut viromes comprise a mixture of virulent and temperate phages, and lifestyle predication based solely on metagenomic data remains uncertain. In contrast, individuals with Crohn’s disease have been reported to display increased abundance of phages carrying temperate-associated markers, alongside reduced virome stability, a pattern that mirrors shifts observed in corresponding bacterial communities.^26^ Similar observations have been reported in studies of colorectal cancer and inflammatory bowel disease, where enrichment of temperate phages has been associated with depletion of their bacterial hosts.^27^ ^,^ ^28^ While these findings highlight strong correlations between phage ecology and disease, causal relationships remain unresolved, underscoring the need for targeted studies using well-characterized phage‒host systems.

Emerging evidence indicates that the composition of the gut virome is also altered in metabolic disorders, including obesity and metabolic syndrome. Metagenomic studies have demonstrated disease-associated shifts in specific phage groups, including crAss-like phages, with phage abundance correlating with bacterial taxa and host metabolic parameters.^29^ ^,^ ^30^ Experimental studies further suggest that phages can actively modulate the structure of the gut ecosystem. For example, fecal virome transplantation has been shown to induce sustained changes in bacterial community composition and improve glucose tolerance in obese mouse models, supporting a functional role for phages in shaping gut microbiota–host metabolic interactions.^31^ Together, these findings extend the association between virome perturbation and disease beyond inflammatory conditions, but the mechanisms by which phages influence microbial community function and host metabolism remain poorly resolved.

To date, no lytic phage infecting *F. prausnitzii* has been described. However, genomic analysis of 15 *F. prausnitzii* strains has identified 18 complete prophages, which are proposed to belong to eight distinct genera, six of which appear to be specific to *F. prausnitzii.* ^32^ Notably, prophages from all but one of these genera were more abundant in individuals with IBD compared to healthy controls, coinciding with reduced *F. prausnitzii* abundance in disease samples.^32^ This observation suggests that prophage dynamics may influence *F. prausnitzii* populations in diseased states. Comparable investigations involving lytic phages have not been possible, largely because of the absence of isolated and characterized lytic *Faecalibacterium* phages. This represents a significant gap in the current understanding of phage‒host interactions involving this key gut bacterium. Given the well-established association between reduced *F. prausnitzii* abundance and multiple disease states, characterizing phages that infect this organism may provide insight into the ecological mechanisms that influence its abundance and contribute to dysbiosis-associated changes in the gut microbial communities. Furthermore, the availability of cultured phages infecting *F. prausnitzii* provides an experimental framework to investigate whether the reduction in *F. prausnitzii* populations contributes to disease development, or whether these reductions arise as a consequence of disease-associated changes within the gut environment. Such studies may help disentangle cause and effect relationships that cannot be resolved through observational microbiome studies alone.

In this study, we report the isolation of the first lytic *Faecalibacterium prausnitzii* phage vB_Fpr_FP01 (hereafter FP01). FP01 was characterized using Transmission Electron Microscopy (TEM), and its genome was sequenced and analyzed using comparative genomic and phylogenetic approaches. Our analyses reveal genomic similarity to uncharacterized phage sequences recovered from human gut microbiome studies, providing foundational insights into lytic phages infecting *F. prausnitzii* and enabling future investigations into phage-mediated lysis, host susceptibility, and the ecological and physiological roles of phages within the gut environment.

## Methods

### Bacterial strains and growth conditions

*Faecalibacterium prausnitzii* (strain APC924/119) culture was obtained from the German Collection of Microorganisms and Cell Culture and was originally isolated from human feces. This bacterium was grown in YCFA media (with 12 g 1^−1^ for agar plates). Media components were sourced from Oxoid, Sigma–Aldrich, and Thermo Fisher Scientific, Australia. Prior to use, all media were deoxygenized for 24 h by incubating in an anaerobic chamber with an internal temperature of 37°C and an atmosphere of 80% N_2_, 10% H_2_, and 10% CO_2_. Plate cultures were grown and incubated within the chamber. Broth cultures were inoculated within the anaerobic environment, removed from the chamber, and propagated using the chi bio platform.^33^ This automated platform, set to 37 °C with a 0.3 stirring rate, allowed for constant agitation and quantification of *F. prausnitzii* growth through OD measurements.

### Isolation and enrichment of FP01

Various environmental samples, including mouse feces and activated sludge, were used to screen for putative lytic phages. These samples were suspended in 10 mL of milliQ water, vortexed, centrifuged at 6,000 × g for 10 min, and filtered through a 0.22-µm pore filter to remove bacteria and associated debris. To enrich for any putative phages, under anaerobic conditions, 1 mL of this environmental sample and 1 mL of active *F. prausnitzii* culture were added to 28 mL of sterile YCFA broth. The samples were incubated at 37 °C for 48 h using the Chi Bio platform (continuous stir set at a rate of 0.3). After propagation, the samples were centrifuged at 6,000 × g for 10 min and filtered through a 0.22-µm pore filter to remove the host bacteria. Within the anaerobic chamber, 30 µL of filtrate was spotted on *F. prausnitzii* lawn. The plates were incubated for 48  h and inspected for the presence of plaques or disturbance. Growth disturbance was observed, and this region was plugged to prevent further propagation.

### Transmission electron microscopy (TEM)

A copper grid (ProSciTech) coated with carbon and Formavar was exposed to glow discharge treatment for 60 s. Subsequently, 10 µL of phage filtrate was loaded and allowed to adhere for 60 s. Excess filtrate was removed with filter paper. The grid was washed with 10 µL of Milli-Q water, and excess water was also removed with filter paper. This step was repeated three times. The grid was then negatively stained with 10 µL of 3% (w/v) uranyl acetate. Excess stain was removed immediately with filter paper. The grid was air-dried for 30 min, and the samples were imaged using a JEM-2100 (JEOL) electron microscope. ImageJ (Fiji) v1.53c was used to measure the capsid and tail lengths.

### Phage DNA extraction and sequencing

To remove contaminating host material, 1 mL of phage filtrate was treated with 10 µg mL^−1^ DNase I, 10 µg mL^−1^ RNase A, and 5 mM MgCl_2_, and incubated for 1 h at 37 °C. Phage virions were precipitated with 10% (w/v) polyethylene glycol (PEG) and 6% (w/v) NaCl and incubated at 4 °C overnight. The sample was centrifuged at 12,000 × g for 10 min before the supernatant was discarded, and the pellet was resuspended in 100 μL of MQ water. To break down the phage capsid and release the DNA, 5 μL of NaCl (1 M), 5 μL of SDS (10%), 4 μL of EDTA (500 mM), and 1 μL of Proteinase K [10 mg/mL] were added, and the sample was incubated at 55 °C for 1 h. Phage DNA was precipitated by adding a 1:1 volume (200 μL) of phenol/chloroform/isoamyl alcohol solution to the cooled sample. This mixture was briefly vortexed and centrifuged at 12,000 × g for 3 min. The middle aqueous layer was transferred to a new tube with an equal volume of isopropanol and incubated for 1 h at −20 °C. The sample was then centrifuged at 16,000 × g for 10 min, and the supernatant was discarded. The pellet was then washed using fresh 70% ethanol before being dried in the Savant DNA 120 Speedvac concentrator (Thermo Scientific). The pellet was resuspended in 40 μL of Milli-Q water and stored at 4 °C.

The isolated phage DNA (<100 ng) was prepared using the NEBNext Ultra II DNA Library Prep Kit (NEB). Subsequently, whole-genome sequencing on an Illumina MiSeq v2 300-cycle kit with 150-bp paired-end reads was completed. The raw data were filtered using fastp (Version 0.23.4) and the genome was assembled using Unicycler (Version 0.5.0), both using default settings.^34^ ^,^ ^35^

### Genome annotations

The FP01 genome was uploaded to Geneious Prime v9.5.5, and genes were predicted by Glimmer3.^36^ Putative open reading frames (ORFs) were manually confirmed and annotated using amino acid sequence similarity through pBLAST analysis and compared to the GenBank database. The programs PhageAI and BACPHILP (BACterioPHage Lifestyle Predictor) were used to detect lysogeny-associated proteins and predict the lifestyle of the phage.^37^ ^,^ ^38^ PhageTerm v1.0.12 analysis was used to determine genome termini and predict the mode of DNA packaging.^39^ To screen for tRNA molecules, the genome was subjected to ARAGORN.^40^

### Genome comparisons

To investigate the genomic similarity of FP01 to other phages, phages of interest were downloaded from the GenBank database. The FASTA sequences were collated and analyzed using VIRIDIC v1.0.^41^ Additionally, a whole Genome BLAST Distance Phylogeny (GBDP) tree was constructed using VICTOR web software.^42^ The d_4_ distance value formula was used when comparisons contained sequences that were incomplete, whereas the d_0_ distance value formula was used when whole-genome sequences were analyzed. Trees were visualized in ITOL v6.^43^ The whole-genome map alignment was generated using the Clinker web software.^44^ Proteome phylogenetic analysis was performed using ViPTree.^45^ The phage articulate network was generated using vContact2.^46^ The FASTA file of FP01 was concatenated with >22,900 phage genomes from the INPHARED database, and combined with tsv and amino acids files, which were used to assign and map the protein sequences for input into vContact2.^47^ The output network file was visualized using Cytoscape v3.9.2 using the global map layout. An edge-weighted spring-embedded model layout was used to examine the smaller group of phages.

## Data availability

The complete genome sequence of the phage FP01 is available in GenBank under accession number PX512812.

## Results

### Isolation and structural characterization of *Faecalibacterium prausnitzii* phage, vB_Fpr_FP01

**Figure 1.**
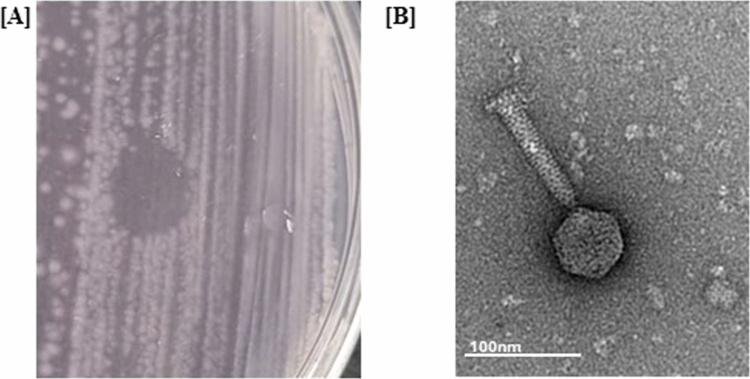
(A) Lytic activity of FP01 on *F.* *prausnitzii* lawn. (B) Transmission electron microscopy images of FP01 virions. The image reveals an icosahedral shaped head and long tail structure.

Activated sludge samples were collected from a local wastewater treatment plant and filtered through a 0.22-µm pore size membrane to remove bacterial cells. An aliquot (1 mL) of the resulting filtrate was added to an actively growing culture of *F. prausnitzii* for phage enrichment. Following incubation, the enrichment was screened for phages infecting *F. prausnitzii* using plaque assays performed on anaerobically incubated bacterial lawns. Zones of growth disturbance were observed on the lawns, indicative of phage activity, and a single entity was isolated and propagated as a putative lytic phage (Figure 1A). The phage was purified through three successive rounds of plaque isolation prior to structural analysis. The availability of only a single *F. prausnitzii* isolate precluded assessment of the host range of FP01 against other representatives of the species. Purified virions were visualized by Transmission Electron Microscopy (TEM) following negative staining with 2% uranyl acetate. TEM analysis revealed that FP01 possesses a myovirus-like morphology, characterized by an icosahedral capsid measuring approximately 61.9 ± 2.3 nm in length × 63.2 ± 2.9 nm in width, and a contractile striated tail approximately 116.9 ± 5.4 nm in length × 20.2 ± 1.5 nm in width (Figure 1B; Supplementary material S1).

### *F. prausnitzii* phage vB_Fpr_FP01 has a unique genome sequence

**Figure 2.**
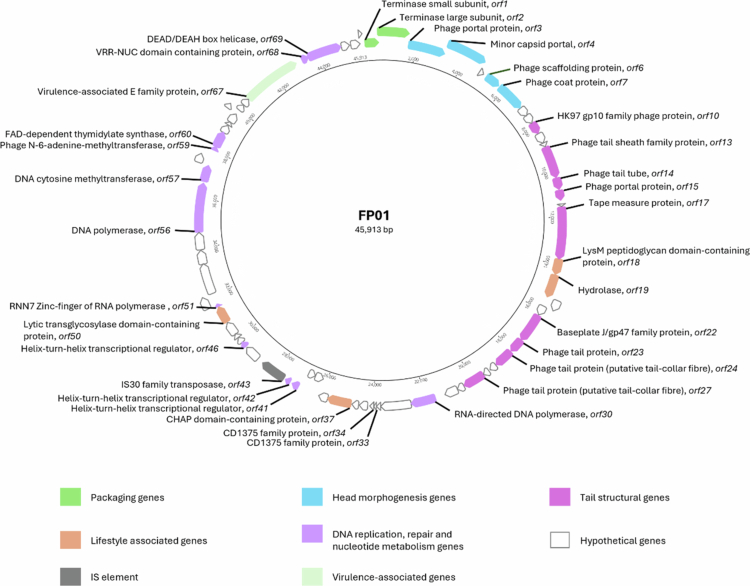
Circular genome map of FP01. ORFs that encode proteins of known function are numbered and labeled with the protein name. Genes are grouped in accordance with the color key at the bottom of the figure: packaging genes (solid green), head morphogenesis genes (solid blue), tail structural genes (solid pink), lifestyle-associated genes (solid orange), DNA replication, repair, and metabolism (solid purple), IS-element (solid gray), and hypothetical genes (gray outlined).

Phage FP01 was isolated, sequenced using Illumina technology, and assembled into a single contig with Unicycler, which was confirmed by Bandage visualization (see Supplementary Figure S2). The complete genome is 45,913 bp in length with a GC content of 57.6%, which is comparable to that of *F. prausnitzii* genomes, which range between 56.3% and 57.2%. The FP01 genome is circularly permuted, and no tRNA genes were detected. Genome annotation using Glimmer3, followed by manual curation, revealed 72 putative open reading frames (ORFs). BLASTp analysis indicated that 33 ORFs encode hypothetical proteins with no assigned functions (Figure 2; Table 1). The remaining 39 ORFs are organized into functional modules associated with DNA packaging, virion morphogenesis, tail structure, DNA replication, metabolism, and repair.

**Table 1.** Summary of ORF and gene products of FP01.

ORF	No. of amino acids	Predicted function (conserved domain)^a^	Match^b^	Percent identity	E-value
1	188	Terminase small subunit (pfam03592)	Terminase small subunit, *F. prausnitzii*	100	1e-133
2	404	Terminase large subunit (COG1783)	PBSX family phage terminase large subunit, *F. prausnitzii*	99.75	0
3	506	Phage portal protein (pfam05133)	Phage portal protein, *F. prausnitzii*	100	0
4	526	Minor capsid protein (COG558)	Phage head morphogenesis protein, *F. prausnitzii*	100	0
5	63	Unknown	*F. prausnitzii*	100	5e-38
6	216	Phage scaffolding protein (pfam06810)	Phage scaffolding protein, Oscillospiraceae	100	2e-151
7	392	Phage coat protein	Phage coat protein, *F. prausnitzii*	100	0
8	134	Unknown (cd08055)	*F. prausnitzii*	100	2e-91
9	124	Unknown	*F. prausnitzii*	100	4e-85
10	165	HK97 gp10 family phage protein (pfam04883)	HK97 gp10 family phage protein, *F. prausnitzii*	100	3e-117
11	141	Unknown	Eubacteriales	100	8e-100
12	61	Unknown	Eubacteriales	100	1e-32
13	440	Phage tail sheath family protein (pfam04984) (pfam17482) (pfam17481)	Phage tail sheath family protein, *F. prausnitzii*	100	0
14	159	Phage tail tube protein (pfam09393)	Phage tail tube protein, Eubacteriales	100	3e-114
15	142	Phage portal protein (pfam08890)	Phage tail assembly chaperone, *F. prausnitzii*	100	5e-90
16	38	Unknown	*Caudoviricetes*	97.30	9e-16
17	700	Tape measure protein (pfam20155)	Tape measure protein, *F. prausnitzii*	100	0
18	215	LysM peptidoglycan domain-containing protein (COG1652)	LysM peptidoglycan domain-containing protein, Eubacteriales	100	2e-153
19	322	Hydrolase (COG3500)	XkdQ/YqbQ family protein, *F. prausnitzii*	100	0
20	131	Unknown (pfam10844)	*Caudovircetes*	99.31	4e-101
21	137	DUF2634 domain-containing protein (pfam10934)	DUF2634 domain-containing protein, *F. prausnitzii*	100	4e-94
22	407	Baseplate J/gp47 family protein (pfam04865)	Baseplate J/gp47 family protein, *F. prausnitzii*	100	0
23	207	Phage tail protein (pfam10076)	Putative phage tail protein, Eubacteriales	100	2e-148
24	265	Phage tail protein (pfam12571)	Putative phage tail protein, Oscillospiraceae	100	0
25	101	Unknown	Oscillospiraceae	100	2e-65
26	103	Unknown	*F. prausnitzii*	100	3e-52
27	299	Phage tail protein (pfam03406)	Phage tail protein, Oscillospiraceae	100	0
28	93	Unknown	Oscillospiraceae	100	2e-58
29	184	Unknown (cd16376)	Oscillospiraceae	100	1e-134
30	342	RNA-directed DNA polymerase (cd1646)	RNA-directed DNA polymerase, *F. prausnitzii*	100	0
31	407	DUF6273 domain-containing protein (pfam19789)	DUF6273 domain-containing protein, *F. prausnitzii*	100	0
32	63	Unknown	Eubacteriales	100	6e-35
33	38	CD1375 family protein (NF040910)	CD1375 family protein, Eubacteriales	100	1e-15
34	39	CD1375 family protein (NF04910)	CD1375 family protein, Oscillospiraceae	100	5e-29
35	113	Unknown	*F. prausnitzii*	100	2e-75
36	93	Unknown	Oscillospiraceae	100	2e-60
37	326	CHAP domain-containing protein (pfam05257)	CHAP domain-containing protein, *F. prausnitzii*	100	0
38	125	Unknown	Oscillospiraceae	100	1e-81
39	127	Unknown	*F. prausnitzii*	100	4e-89
40	79	Unknown	*F. prausnitzii*	100	7e-50
41	95	Helix-turn-helix transcriptional regulator (COG1396)	Helix-turn-helix transcriptional regulator, Oscillospiraceae	100	3e-60
42	79	Helix-turn-helic transcriptional regulator (COG1396)	Helix-turn-helic transcriptional regulator, *F. prausnitzii*	100	2e-47
43	349	IS30 family transposase (COG2826)	IS30 family transposase, *F. prausnitzii*	100	0
44	42	Unknown	*Caudoviricetes*	92.68	2e-19
45	208	Unknown	*F. prausnitzii*	100	6e-153
46	84	Helix-turn-helix domain-containing protein (COG2826)	Helix-turn-helix domain-containing protein, Oscillospiraceae	100	7e-51
47	69	Unknown	Bacteriophage sp	98.53	7e-39
48	58	Unknown	Oscillospiraceae	100	5e34
49	194	Unknown	*F. prausnitzii*	100	2e-142
50	238	Lytic transglycosylase domain-containing protein (pfam01464)	Lytic transglycosylase domain-containing protein, *F. prausnitzii*	100	2e-174
51	40	RNN7 Zinc-finger of RNA polymerase I-specific TFIIB Rrm7 (PRK14714)	RNN7 Zinc-finger of RNA polymerase I-specific TFIIB Rrm7, *Caudovircetes*	100	3e-19
52	163	Unknown	*F. prausnitzii*	100	5e-103
53	413	DUF2800 domain-containing protein (pfam10926)	DUF2800 domain-containing protein, *F. prausnitzii*	100	0
54	188	Unknown	*F. prausnitzii*	100	4e-138
55	232	DUF2815 family protein (pfam10991)	DUF2815 family protein, *F. prausnitzii*	100	3e-166
56	667	DNA polymerase (cd08642)	DNA polymerase, *F. prausnitzii*	100	0
57	225	DNA cytosine methyltransferase	DNA cytosine methyltransferase, *F. prausnitzii*	100	4e-167
58	104	Unknown (COG1439)	*F. prausnitzii*	100	5e-72
59	86	Phage *N*-6-adenine-methyltransferase (pfam05869)	DNA *N*-6-adenine-methyltransferase, *Caudovircetes* sp	76.47	4e-38
60	219	FAD-dependent thymidylate synthase (COG1351)	FAD-dependent thymidylate synthase, *F. prausnitzii*	100	3e-158
61	113	DUF6378 domain-containing protein (pfam19905)	DUF6378 domain-containing protein, *F. prausnitzii*	100	5e-77
62	50	Unknown	*F. prausnitzii*	97.96	3e-27
63	136	Unknown	*F. prausnitzii*	100	9e-95
64	71	Unknown	Oscillospiraceae	100	5e-43
65	97	Unknown	*F. prausnitzii*	100	1e-62
66	98	Unknown (cd11539)	Oscillospiraceae	100	3e-64
67	801	Virulence-associated E family protein (COG5545)	Virulence-associated E family protein, *F. prausnitzii*	100	0
68	94	VRR-NUC domain-containing protein (cd22365)	VRR-NUC domain-containing protein, Oscillospiraceae	100	2e-61
69	463	DEAD/DEAH box helicase (cd18013)	DEAD/DEAH box helicase, Oscillospiraceae	100	0
70	113	Unknown	*F. prausnitzii*	100	2e-74
71	127	Unknown	*F. prausnitzii*	100	4e-87
72	55	Unknown	*Myoviridae*	82.93	6e-16

^a^ Predicted function based on BLASTp analysis and domain accession number included in brackets if present.

^b^ The most closely related gene (if named) and the name of the organism.

The genome was oriented with the small terminase subunit gene (*terS*) designated as the first open reading frame, followed by the large terminase subunit gene (*terL*) and the portal protein gene (*orf3*). These annotations were assigned based on conserved protein domains and amino acid sequence homology. Illumina sequencing generated 128,074 raw reads, of which 126,914 reads (99.1%) passed quality filtering with fastp, yielding 14.03 Mb of high-quality sequence data. Based on an assembled genome size of 45,913 bp, the theoretical sequencing depth was approximately 300×. However, read mapping revealed substantially lower effective genome coverage (~68×), with 25.1% of the reads failing to align to the reference genome, indicating uneven sequence coverage across the assembly.

PhageTerm analysis was unable to confidently identify genome termini using Li’s method. No significant termini peaks were detected (forward/reverse = 0/0), and the calculated Li’s method parameters (R1 = 19, R2 = 1, and R3 = 1) were consistent with the absence of clearly defined genome ends. This pattern is compatible with either a circular genome or a circularly permuted genome exhibiting terminal redundancy. However, because the effective coverage (<200×) and substantial proportion of reads did not align to the assembled genome, the packaging strategy and genome termini of FP01 could not be determined with confidence (Supplementary Figure S3).

BLASTp analysis of TerL revealed high similarity (99.75%) to a PBSX family terminase large subunit from *F. prausnitzii* and contained the conserved XtmB domain (COG1783). Despite similarity to PBSX-like terminases, which have historically been associated with phage-derived bacteriocin, FP01 exhibited clear lytic activity on bacterial lawns, and intact virions were observed by transmission electron microscopy.^48^ Additionally, the genome encodes a full complement of structural, replication, and lysis-associated genes, supporting FP01 being a functional lytic bacteriophage.

Immediately downstream of the packaging genes, a cluster of six ORFs encodes proteins involved in capsid morphogenesis. These include the minor capsid protein (Orf4; COG5585), a hypothetical protein with a gp20-like domain (Orf5), a scaffolding protein (Orf6), the phage coat protein (Orf7), a putative head–tail connector protein (Orf8), and a head closure protein (Orf9). These proteins are consistent with a typical tailed phage capsid assembly module.

Genes encoding tail structural components follow the head morphogenesis module. This region contains eight ORFs, including a tail component protein (Orf10; pfam04883), tail sheath protein (Orf13; pfam17481, pfam04984, and pfam17482), tail chaperone protein (Orf14; DUF2001), a tail assembly protein (Orf15; pfam08890), and the tape measure protein (Orf17; pfam20155), which is the largest predicted protein in the genome.^49^ Three ORFs within this cluster (*orf11, orf12* and *orf16*) encode proteins of unknown function.

FP01 also encodes several proteins involved in nucleotide metabolism and genome maintenance. These include an FAD-dependent thymidylate synthase (*orf60*), two predicted DNA methyltransferases (*orf57 and orf59*), a virus-type replication-repair nuclease (*orf68*), and a DEAD/DEAH box helicase gene (*orf69*). In addition, three helix-turn-helix transcriptional regulators (*orf41, orf42*, and *orf46*) and a putative RNA-directed DNA polymerase/reverse transcriptase (*orf30*) were identified. The presence of these auxiliary metabolic and regulatory genes suggests that FP01 encodes functions that may enhance nucleotide availability and replication efficiency during infection. This phenomenon has been previously reported with phages enriching metabolites involved in amino acid synthesis or encoding nucleotide metabolism enzymes.^50^ ^,^ ^51^

vB_Fpr_FP01 is predicted to exhibit a lytic lifestyle that has horizontally acquired genes from its host. FP01 was isolated following the observation of localized growth disturbance on *F. prausnitzii* bacterial lawns, consistent with lytic activity (Figure 1A). To assess the replication strategy of FP01, two independent lifestyle prediction tools were applied. BACPHLIP, which predicts phage lifestyle based on the presence of lysogeny-associated protein domains,^37^ classified FP01 as lytic with a virulent score of 0.79 compared to a temperate score of 0.21. Consistent with this prediction, genome annotation did not identify an integrase, recombinase, excisionase, CI-like repressor, or other genes typically associated with lysogeny.

In parallel, FP01 was analyzed using PhageAI, which predicts phage lifestyle from proteome similarity.^38^ In contrast to BACPHLIP, PhageAI classifies FP01 as temperate with a confidence of 89.59%. Inspection of the phylogenetic clustering indicated that FP01 grouped with phages encoding PBSX-like terminase subunits, suggesting that similarity within this protein family may have influenced its classification. Notably, the closest relative identified in the PhageAI was itself annotated as a virulent phage. Despite these conflicting predictions, the absence of lysogeny-associated genes and identifiable att-site motifs supports a lytic replication strategy. This interpretation is further supported by plaque formation and virion production during host infection.

Genome annotation identified multiple putative lysis-associated proteins, including a LysM peptidoglycan binding domain-protein (*orf18*; pfam01476), a hydrolase containing the COG3500 domain (*orf19*), a cysteine, histidine-dependent amidohydrolase/peptidase (CHAP) domain-containing protein (*orf37*; pfam05257), and a lytic transglycosylase (*orf50*). Together, these proteins are predicted to target the bacterial peptidoglycan layer through distinct enzymatic mechanisms, providing a plausible basis for host cell lysis.^52^ ^,^ ^53^ Interestingly, these genes were dispersed throughout the genome rather than organized within a discrete lysis module. To further investigate the potential for lysogeny, 25 complete *F. prausnitzii* genomes retrieved from GenBank were screened for FP01-related sequences. Fifteen genomes contained annotated phage-associated genes, including holins, terminases, and capsid proteins, whereas the remaining genomes lacked phage annotations. However, no significant similarity to FP01 was detected in any strain (Supplementary Table 1), suggesting that it is not maintained as an integrated prophage and shares limited homology with previously identified prophages in this species. Similarly, no significant similarity was detected in the genomes of *F. prausnitzii* APC918/95b (CP030777.1) or *Faecalibacterium duncaniae* A2165 (NZ_CP022479.1). The FP01 genome also encodes genetic elements commonly associated with horizontal gene transfer. An insertion sequence homologous to IS30 was identified (*orf43)*, which shares similarity with IS-elements found in several human gut-associated bacterial genera, including *Faecalibacterium* (accession number: WP_112121027)*, Dysosmobacter* (accession number: WP_325227381.1), and *Vescimonas* (accession number: WP_421128919.1). In addition, a gene encoding a virulence-associated E family protein (*orf67*; pfam05272) was identified. Members of the VirE superfamily are commonly associated with bacterial virulence, although the function of this protein in FP01 remains unknown.

### Whole-genome comparisons and classification of FP01

FP01 was isolated from wastewater; however, its host strain (*F. prausnitzii* strain APC924/119) was originally isolated from human feces.^54^ To explore whether related viral sequences occur in gut-associated environments, the FP01 genome was compared against the Unified Human Gut Virome (UGHV) database, a comprehensive collection of 873,995 viral genomes derived from 12 independent studies.^55^ This analysis identified 71 UGHV viral sequences sharing similarity with FP01 (Supplementary Table 2). Although the sequence similarity was high (up to 99.72%), the alignments were generally partial, and relatively few closely related sequences were identified, likely reflecting the fragmented nature of metagenome-derived viral genomes. These findings indicate that FP01-related viral sequences are represented within current human gut virome datasets, although the hosts of these sequences remain unknown. Additionally, FP01 was compared against the ICTV TaxaBlast database, which contains more than 23,000 classified viral genomes. No significant similarity was detected to any ICTV-classified viral genome, further supporting the distinct nature of FP01.

To classify FP01 and assess its relatedness to other phages, whole-genome similarity and phylogenetic analysis were performed. The FP01 genome shares detectable nucleotide homology with three complete genomes deposited in GenBank. These genomes, named isolate 3552_106289 (accession number OP075608.1), isolate 2423_15267 (accession number OP074578.1), and isolate 3584_27091 (accession number OP075639.1), were recovered from a human gut virome metagenomic study conducted in Japan (BioProject: PRJNA862966), and their bacterial hosts remain unknown.^56^

**Figure 3.**
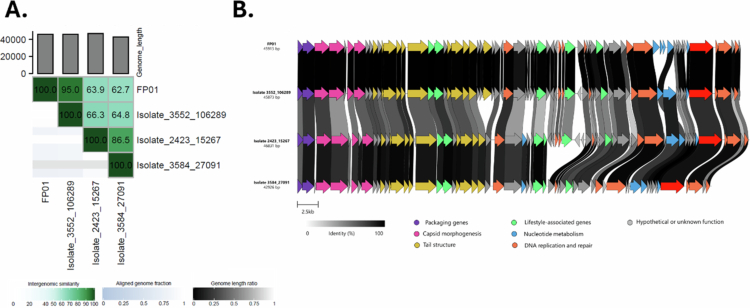
Whole-genome investigation of FP01 and the three complete related phages identified through BLASTn, isolate 3552_106289 (accession number OP075608.1), isolate 2423_15267 (accession number OP074578.1), and isolate 3584_27091 (accession number OP075639.1). (A) A heat map was generated to demonstrate the intergenomic similarities between FP01and related phages. (B) Whole genome map alignments of phages. Sequences were aligned from the small terminase gene, shared percentage identity is indicated by the greyscale bar and key gene groupings are categorised by colours.

Intergenomic similarities were quantified using VIRIDIC.^41^ Using established nucleotide similarity thresholds of 95% and 70% to delineate species and genera, respectively, FP01 shared 95.0% similarity with isolate 35552_106289 and was assigned the same genus (Figure 3A). In contrast, isolates 2423_15267 and isolates 3584_27091 share 63.9% and 62.7% similarity with FP01, respectively, placing them within a separate genus (Figure 3A; Supplementary Tables 3 and 4). All four phages were classified as distinct species.

Whole-genome alignment analysis was conducted to examine the genomic synteny between FP01 and these related phages (Figure 3B). The alignments were anchored at the small terminase subunit gene and revealed a conserved overall genome architecture across all four phages. FP01 exhibited the highest sequence identity and synteny with isolate 3552_106289, with differences primarily confirmed for genes encoding hypothetical proteins. Notably, isolate 3552_106289 encodes a DNA cytosine methylase containing the COG0270 domain that is absent from the FP01 genome. Reduced synteny and increased alignment gaps were observed between FP01 and isolate 2423_15267 and isolate 3584_27091, consistent with the VIRIDIC-based genus assignments.

To further investigate the phylogenetic placement of FP01, a proteome-based phylogenetic analysis was performed using ViPTree, with the three most closely related phage genomes identified in GenBank included in the analysis. The resulting cladogram placed FP01 within a clade containing these related phages, none of which had an identified host (Figure 3). This clade was distinct from neighboring clusters, which largely comprised phages infecting common bacterial hosts, as indicated by the colored boxes. These findings suggest the FP01 and its closest relative represent a distinct lineage within the dataset, sharing only limited proteomic similarity with other classified phages.

**Figure 4.**
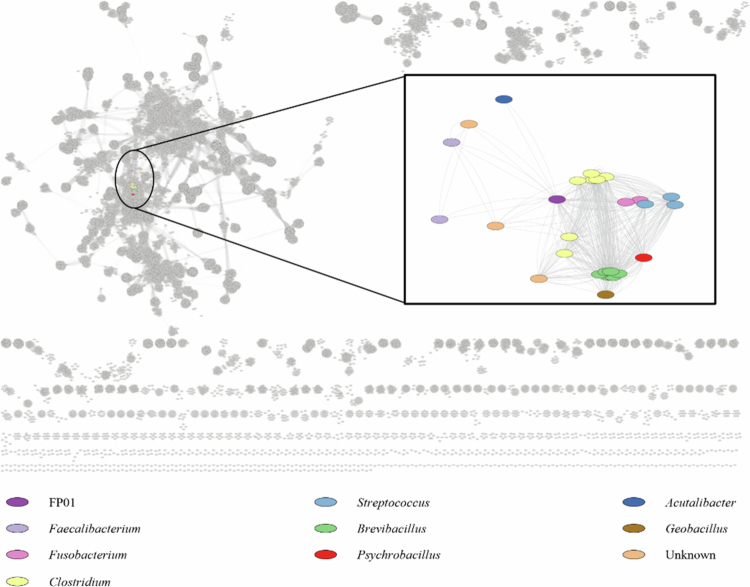
Gene sharing of FP01 and phages from the INPRARED database. A zoomed-in phage reticulate network of FP01 is shown in the panel on the left, illustrating the relatives using an edge-weight spring-embedded layout model. Each circle is a node that represents a viral genome, and gene sharing is represented by the edges or gray lines connecting nodes. Furthermore, nodes are colored to indicate the bacterial host genera.

To further investigate gene-sharing relationships, vConTACT2 was used in conjunction with the INPHARED database to construct a gene-sharing network (Figure 4).^46^ ^,^ ^47^ As of Dec 2024, the database comprises 25,692 phage genomes and 2,434,906 gene-sharing interactions. In the global network, FP01 clustered within a larger interconnected phage group. To improve visualization, nearest-neighbor genomes were extracted, and an edge-weighted spring layout was applied. This FP01-specific network comprised twenty-six phages infecting a range of bacterial genera, including *Clostridium, Fusobacterium, Streptococcus, Brevibacillus, Psychrobacillus, Actualibacter,* and *Geobacillus* (indicated by the colors in Figure 4) and included 406 gene sharing events.

**Figure 5.**
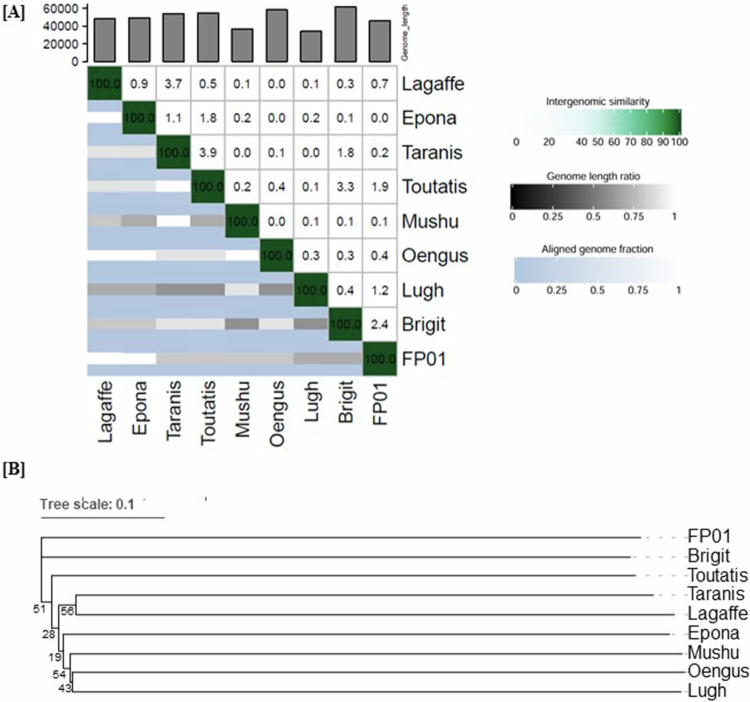
Whole-genome comparison and phylogenetic relationships between FP01 and *F. prausnitzii* prophages. (A) A heat map was generated to display the intergenomic similarities between FP01 and *F. prausnitzii* prophages. (B) Whole-genome (nucleotide) phylogenetic tree using the GBDP d0 formula was completed on FP01 and prophages.

Within this network, FP01 shared genes with two previously described *F. prausnitzii* prophages, Lagaffe and Brigit (Figure 4; Lilac colored nodes). Cornault et al. (2019) identified eighteen *F. prausnitzii* prophages from fifteen bacterial genomes and proposed eight phage genera based on structural gene classification.^32^ To assess the relatedness of FP01 to these prophages, VIRIDIC analysis was performed using available complete prophage sequences. No intergenomic clustering was observed, with all phages classified as distinct genera (Figure 5A). Prophage Brigit shared the highest similarity with FP01 (2.4%), followed by Toutatis (1.9%) and Lugh (1.2%), while no shared sequence was detected between FP01 and Epona. Genome-scale phylogenetic analysis using VICTOR supported these findings, resolving FP01, Brigit, and Toutatis into distinctly related clades (Figure 5B).^32^

## Discussion

In this study, we report the isolation and characterization of FP01, the first lytic bacteriophage infecting *Faecalibacterium prausnitzii*. Virion morphology revealed a myovirus-like architecture consistent with members of the class *Caudoviricetes*, while genomic analysis demonstrated that FP01 possesses a highly novel genome with limited similarity to previously characterized phages. Although FP01 does not cluster closely with known isolates, it shares detectable homology with phage genomes identified in a large-scale human gut virome study (BioProject: PRJNA862966), which analyzed the dsDNA virome from 4,198 participants across a range of demographic, lifestyle, and health variables.^56^ Notably, the phage genomes recovered in that study were metagenome-assembled and could not be confidently assigned to bacterial hosts, thereby limiting interpretation of their ecological roles within the gut microbiome.

The lack of experimentally validated host assignments remains a major limitation in gut virome research. Unlike bacteria, phages lack a universally conserved marker gene analogous to the 16S rRNA gene, complicating both taxonomic classification and host predation.^57^ While single-marker genes such as the large terminase subunit (*terL*) are useful for comparative analyses within defined lineages, they are not broadly applicable across phage diversity.^58^ As a result, many phage genomes identified through metagenomics remain disconnected from their bacterial hosts, hindering efforts to understand how phages shape microbial community structure, population stability, and metabolic function within the gut ecosystem.

Alterations to gut microbial communities have been consistently associated with a range of inflammatory and metabolic conditions, often involving reproducible shifts in keystone taxa.^59^ ^,^ ^60^ *F. prausnitzii* is a prominent butyrate-producing anaerobe with well-established anti-inflammatory properties, and reductions in its abundance have been reported across multiple disease contexts, frequently correlating with increased markers of low-grade inflammation.^61-63^ A decrease in *F. prausnitzii* and other butyrate-producing bacteria has also been observed in individuals with hypertension, alongside reduced levels of microbiota-derived anti-inflammatory metabolites. Despite these consistent associations, the drivers of *F. prausnitzii* depletion remain unresolved and are likely to involve a complex interplay of host physiology, environmental factors, and microbial interactions, rather than a single causative mechanism. Recent studies have demonstrated that some phage groups decrease, while others expand in diversity in obesity, suggesting that specific phage lineages may play distinct roles in metabolic disease and contribute to shaping microbial community structure.^29^ ^,^ ^30^ Additionally, fecal virome transplantation studies have demonstrated that phages can modulate important bacterial taxa and influence host metabolic functions.^31^ However, such studies provide limited mechanistic insight and do not resolve the contribution of specific phage‒host interactions, particularly those involving key gut commensals. Therefore, the isolation of a lytic *F. prausnitzii* phage provides a tractable system to investigate how phage predation influences the abundance and ecology of this important anti-inflammatory bacterium. Such studies may help determine whether a reduction in *F. prausnitzii* contributes to disease-associated dysbiosis or arises as a consequence of disease-related changes in the gut ecosystem.

FP01 was isolated from wastewater rather than directly from the human gastrointestinal tract, whereas the host *F. prausnitzii* strain was originally isolated from human feces.^54^ While wastewater environments sustain many gut-derived microorganisms, they differ substantially from the human gut in terms of physicochemical conditions, microbial communities, nutrient availability, and selection pressures. These distinct niches influence microbial ecology and may affect phage genomics, host range, and infection dynamics.^64^ For instance, phages isolated from wastewater may be exposed to a range of environmental stresses and microbial hosts rather than strictly gut-associated phages, potentially influencing their infectivity and evolutionary trajectory.^65^ ^,^ ^66^ Although FP01 is capable of infecting a human gut-derived *F. prausnitzii* strain under laboratory conditions, its infectivity, replication dynamics, and ecological role within the gut environment may differ. Therefore, caution is warranted when extrapolating the relevance of FP01 to in situ gut ecosystems. Nevertheless, the identification of closely related viral sequences in human gut virome datasets suggests that FP01-related phages are not restricted to wastewater environments and may be distributed across both host-associated and environmental niches. However, the hosts of these gut-derived viral sequences remain unknown. Consequently, experimental validation using gut-relevant model systems will be required to determine whether FP01-like phages actively infect *F. prausnitzii* populations in vivo.

The current ICTV framework has moved away from structural characteristic groupings but rather encompasses genome-based taxonomy and phylogenetic relationships. Under these guidelines, tailed double-stranded DNA phages such as FP01 are classified within the class Caudoviricetes. However, FP01 does not cluster closely with any currently recognized genus or family based on available reference genomes, reflecting broader challenges in phage taxonomy whereby the discovery of novel phages continues to outpace formal classification.^67^ This observation is consistent with previous studies demonstrating that many gut-associated bacteriophages remain uncultured and unclassified, forming part of the viral ‘dark matter’ owing to their limited representation in existing databases.^68^ The absence of close taxonomic assignment suggests that FP01 may belong to a distinct and previously uncharacterized lineage within Caudoviricetes. Similar patterns have been reported for phages infecting other gut-associated bacteria, highlighting the substantial unexplored diversity of the human gut virome.^68^ ^,^ ^69^ Accordingly, FP01 adds to the growing body of evidence that a considerable proportion of bacteriophage diversity remains unresolved within current taxonomic frameworks.

Consistent with observations across phage genomics, a substantial proportion of the FP01 genome encodes hypothetical proteins of unknown function. This reflects the broader issue of viral “dark matter” within gut viromes, where many phage genomes lack similarity to annotated sequences in public databases.^70^ ^,^ ^71^ While metagenomic sequencing has greatly expanded the cataloging of gut viral diversity, functional and ecological interpretation remains constrained in the absence of cultured representatives.^72-74^ In this context, the isolation of FP01 provides a critical biological reference that can facilitate more accurate interpretation of *Faecalibacterium*-associated viral contigs identified in metagenomic surveys and support efforts to link viral diversity with microbial function.

Genomic analysis of FP01 further suggests historical genetic exchange between phages and bacterial genomes. FP01 encodes an IS30 insertion sequence element with similarity to those found in several anaerobic bacteria. The incorporation of IS-elements into phage genomes is well documented and reflects the role of phages in horizontal gene transfer within microbial communities. It is therefore plausible that this element was acquired through genetic exchange with the bacterial host, potentially *Faecalibacterium*. FP01 also contains a gene annotated as encoding a virulence-associated E family protein; however, the functional relevance of this gene in the context of a commensal gut bacterium remains unclear and should not be interpreted as evidence of pathogenic potential. Rather, its presence likely reflects shared gene pools and historical exchange within densely populated microbial environments.

Bacteriophages represent an integral but still underexplored component of the gut microbiome. Advances in understanding their ecological roles have been limited by the difficulty of culturing many gut-associated anaerobes, the dependence on plaque-based isolation methods, and the ongoing challenges of phage taxonomy. Although metagenomic studies have identified extensive viral diversity in both healthy and diseased states, the scarcity of experimentally characterized phages linked to key commensal bacteria has constrained mechanistic insight into how phages contribute to microbial stability, resilience, and functional output. Isolation-based studies, therefore, remain essential for complementing sequencing-based approaches and anchoring gut virome research in experimentally tractable systems.^75^

The identification of a lytic phage infecting *F. prausnitzii* provides a new opportunity to investigate phage–host interactions involving a bacterium central to gut health and homeostasis. In controlled experimental models, such phages can be used to explore how viral predation influences *F. prausnitzii* population dynamics, microbial cross-feeding and community structure, and whether phage activity contributes to, or responds to, shifts in the gut environment associated with disease states. In conclusion, this study describes the isolation and genomic characterization of FP01, the first reported lytic phage infecting *F. prausnitzii,* an important anti-inflammatory member of the human gut microbiota. Comparative genomic analysis expands the current understanding of anaerobic gut phage diversity and provides a much-needed biological reference for *Faecalibacterium*-associated viral sequences identified through metagenomics. Although FP01 was isolated from wastewater rather than directly from gut samples, its genomic novelty and relationship to gut virome sequences suggest that related phages are likely to be present within human-associated microbial ecosystems. The availability of FP01 now enables mechanistic studies to dissect phage–host interactions involving a key commensal anaerobe and supports ongoing efforts to connect viral dark matter with microbial ecology and host physiology.

## Supplementary Material

Supplementary Material Supplementary material.docx

## Data Availability

The data that support the findings of this study are openly available in GenBank at https://www.ncbi.nlm.nih.gov/genbank/. The FP01 complete genome sequence is available under accession number PX512812. The three uncharacterized phages sharing the highest similarity to FP01 are available under the BioProject: PRJNA862966 and accession numbers OP075608.1, OP074578.1, and OP075639.1.

## References

[1] Hold GL , Schwiertz A , Aminov RI , Blaut M , Flint HJ . Oligonucleotide probes that detect quantitatively significant groups of butyrate-producing bacteria in human feces. Appl Environ Microbiol. 2003;69:4320–4324. doi: 10.1128/AEM.69.7.4320-4324.2003. 12839823 PMC165216

[2] Kadhim FJ , Aziz ZS , Ibrahim KS . Gut microbiome profiles in colorectal cancer patients in Iraq. Microbiol Res. 2025;16:22. doi: 10.3390/microbiolres16010022.

[3] Ahmadi A , Kouhsari E , Razavi S , Mohamadzadeh N , Besharat S , Vakili MA , Amiriani T . Comparative analysis of dominant gut microbiota in inflammatory bowel disease patients and healthy individuals: a case-control study. New Microbes New Infect. 2025;64:101567. doi: 10.1016/j.nmni.2025.101567. 39991465 PMC11846925

[4] Wu X , Park S . Fecal bacterial community and metagenome function in asians with type 2 diabetes, according to enterotypes. Biomedicines. 2022;10:2998. doi: 10.3390/biomedicines10112998. 36428566 PMC9687834

[5] Ejtahed H-S , Hoseini-Tavassol Z , Khatami S , Zangeneh M , Behrouzi A , Ahmadi Badi S , Moshiri A , Hasani-Ranjbar S , Soroush A , Vaziri F , et al. Main gut bacterial composition differs between patients with type 1 and type 2 diabetes and non-diabetic adults. J Diabetes Metab Disord. 2020;19:265–271. doi: 10.1007/s40200-020-00502-7. 32550175 PMC7270240

[6] Cai M , Lin L , Jiang F , Peng Y , Li S , Chen L . Gut microbiota changes in patients with hypertension: a systematic review and meta‐analysis. J Clin Hypertens. 2023;25:1053–1068. doi: 10.1111/jch.14722. PMC10710550 37853925

[7] Jie Z , Xia H , Zhong S , Feng Q , Li S , Liang S , Liu Z , Gao Y , Zhao H , Zhang D , et al. The gut microbiome in atherosclerotic cardiovascular disease. Nat Commun. 2017;8:845. doi: 10.1038/s41467-017-00900-1. 29018189 PMC5635030

[8] Cui X , Ye L , Li J , Jin L , Wang W , Bao M , Wu S , Geng B , Zhou X , Zhang J , et al. Metagenomic and metabolomic analyses unveil dysbiosis of gut microbiota in chronic heart failure patients. Sci Rep. 2018;8:635. doi: 10.1038/s41598-017-18756-2. 29330424 PMC5766622

[9] Evans SJ , Bassis CM , Hein R , Assari S , Flowers SA , Kelly MB , Young VB , Ellingrod VE , McInnis MG . The gut microbiome composition associates with bipolar disorder and illness severity. J Psychiatr Res. 2017;87:23–29. doi: 10.1016/j.jpsychires.2016.12.007. 27988330 PMC5336480

[10] Jiang H , Ling Z , Zhang Y , Mao H , Ma Z , Yin Y , Wang W , Tang W , Tan Z , Shi J , et al. Altered fecal microbiota composition in patients with major depressive disorder. Brain Behav Immun. 2015;48:186–194. doi: 10.1016/j.bbi.2015.03.016. 25882912

[11] Jiang H-Y , Zhang X , Yu Z , Deng M , Zhao J , Ruan B . Altered gut microbiota profile in patients with generalized anxiety disorder. J Psychiatr Res. 2018;104:130–136. doi: 10.1016/j.jpsychires.2018.07.007. 30029052

[12] Duncan SH , Hold GL , Harmsen HJ , Stewart CS , Flint HJ . Growth requirements and fermentation products of fusobacterium prausnitzii, and a proposal to reclassify it as *Faecalibacterium prausnitzii* gen. Nov., comb. Nov. Int J Syst Evol Microbiol. 2002;52:2141–2146. doi: 10.1099/00207713-52-6-2141. 12508881

[13] Den Besten G , van Eunen K , Groen AK , Venema K , Reijngoud D , Bakker BM . The role of short-chain fatty acids in the interplay between diet, gut microbiota, and host energy metabolism. J Lipid Res. 2013;54:2325–2340. doi: 10.1194/jlr.R036012. 23821742 PMC3735932

[14] Liu J , Tan Y , Cheng H , Zhang D , Feng W , Peng C . Functions of gut microbiota metabolites, current status and future perspectives. Aging Dis. 2022;13:1106. doi: 10.14336/AD.2022.0104. 35855347 PMC9286904

[15] Bäckhed F , Roswall J , Peng Y , Feng Q , Jia H , Kovatcheva-Datchary P , Li Y , Xia Y , Xie H , Zhong H , et al. Dynamics and stabilization of the human gut microbiome during the first year of life. Cell Host Microbe. 2015;17:690–703. doi: 10.1016/j.chom.2015.04.004. 25974306

[16] Tyakht AV , Kostryukova ES , Popenko AS , Belenikin MS , Pavlenko AV , Larin AK , Karpova IY , Selezneva OV , Semashko TA , Ospanova EA , et al. Human gut microbiota community structures in urban and rural populations in russia. Nat Commun. 2013;4:2469. doi: 10.1038/ncomms3469. 24036685 PMC3778515

[17] Barber TM , Kabisch S , Pfeiffer AF , Weickert MO . The effects of the Mediterranean diet on health and gut microbiota. Nutrients. 2023;15:2150. doi: 10.3390/nu15092150. 37432307 PMC10180651

[18] Fouhy F , Guinane CM , Hussey S , Wall R , Ryan CA , Dempsey EM , Murphy B , Ross RP , Fitzgerald GF , Stanton C , et al. High-throughput sequencing reveals the incomplete, short-term recovery of infant gut microbiota following parenteral antibiotic treatment with ampicillin and gentamicin. Antimicrob Agents Chemother. 2012;56:5811–5820. doi: 10.1128/AAC.00789-12. 22948872 PMC3486619

[19] Hooper LV , Littman DR , Macpherson AJ . Interactions between the microbiota and the immune system. Science. 2012;336:1268–1273. doi: 10.1126/science.1223490. 22674334 PMC4420145

[20] Gregory AC , Zablocki O , Zayed AA , Howell A , Bolduc B , Sullivan MB . The gut virome database reveals age-dependent patterns of virome diversity in the human gut. Cell Host Microbe. 2020;28:724–740. e728. doi: 10.1016/j.chom.2020.08.003. 32841606 PMC7443397

[21] Liang G , Bushman FD . The human virome: assembly, composition and host interactions. Nat Rev Microbiol. 2021;19:514–527. doi: 10.1038/s41579-021-00536-5. 33785903 PMC8008777

[22] Sausset R , Petit M-A , Gaboriau-Routhiau V , De Paepe M . New insights into intestinal phages. Mucosal Immunol. 2020;13:205–215. doi: 10.1038/s41385-019-0250-5. 31907364 PMC7039812

[23] Dyson ZA , Brown TL , Farrar B , Doyle SR , Tucci J , Seviour RJ , Petrovski S , Lin B . Locating and activating molecular ‘time bombs’: induction of mycolata prophages. PLoS One. 2016;11:e0159957. doi: 10.1371/journal.pone.0159957. 27487243 PMC4972346

[24] Zuppi M , Hendrickson HL , O’Sullivan JM , Vatanen T . Phages in the gut ecosystem. Front Cell Infect Microbiol. 2022;11:822562. doi: 10.3389/fcimb.2021.822562. 35059329 PMC8764184

[25] Hargreaves KR , Kropinski AM , Clokie MR . Bacteriophage behavioral ecology: how phages alter their bacterial host’s habits. Bacteriophage. 2014;4:e85131. doi: 10.4161/bact.29866. PMC4124054 25105060

[26] Clooney AG , Sutton TD , Shkoporov AN , Holohan RK , Daly KM , O’Regan O , Ryan FJ , Draper LA , Plevy SE , Ross RP , et al. Whole-virome analysis sheds light on viral dark matter in inflammatory bowel disease. Cell Host Microbe. 2019;26:764–778. e765. doi: 10.1016/j.chom.2019.10.009. 31757768

[27] Hannigan GD , Duhaime MB , Ruffin IV , Koumpouras MT , Schloss CC . P. D. diagnostic potential and interactive dynamics of the colorectal cancer virome. mBio. 2018;9:02248–02218. doi: 10.1128/mbio. PMC6247079 30459201

[28] Nishiyama H , Endo H , Blanc-Mathieu R , Ogata H . Ecological structuring of temperate bacteriophages in the inflammatory bowel disease-affected gut. Microorganisms. 2020;8:1663. doi: 10.3390/microorganisms8111663. 33121006 PMC7692956

[29] Cervantes-Echeverría M , Gallardo-Becerra L , Cornejo-Granados F , Ochoa-Leyva A . The two-faced role of crAssphage subfamilies in obesity and metabolic syndrome: between good and evil. Genes. 2023;14:139. doi: 10.3390/genes14010139. 36672880 PMC9858991

[30] Bikel S , López-Leal G , Cornejo-Granados F , Gallardo-Becerra L , García-López R , Sánchez F , Equihua-Medina E , Ochoa-Romo JP , López-Contreras BE , Canizales-Quinteros S , et al. Gut dsDNA virome shows diversity and richness alterations associated with childhood obesity and metabolic syndrome. iSci. 2021;24:102900. doi: 10.1016/j.isci.2021.102900. PMC8361208 34409269

[31] Cervantes-Echeverría M , Jimenez-Rico MA , Manzo R , Hernández-Reyna A , Cornejo-Granados F , Bikel S , González V , Hurtado Ramírez JM , Sánchez-López F , Salazar-León J , et al. Human-derived fecal virome transplantation (FVT) reshapes the murine gut microbiota and virome, enhancing glucose regulation. PLoS One. 2025;20:e0337760. doi: 10.1371/journal.pone.0337760. 41348832 PMC12680211

[32] Cornuault JK , Petit M , Mariadassou M , Benevides L , Moncaut E , Langella P , Sokol H , De Paepe M . Phages infecting *Faecalibacterium prausnitzii* belong to novel viral genera that help to decipher intestinal viromes. Microbiome. 2018;6:65. doi: 10.1186/s40168-018-0452-1. 29615108 PMC5883640

[33] Steel H , Habgood R , Kelly C , Papachristodoulou A . Chi. Bio: an open-source automated experimental platform for biological science research. BioRxiv. 2019:796516.

[34] Chen S , Zhou Y , Chen Y , Gu J . Fastp: an ultra-fast all-in-one FASTQ preprocessor. Bioinformatics. 2018;34:i884–i890. doi: 10.1093/bioinformatics/bty560. 30423086 PMC6129281

[35] Wick RR , Judd LM , Gorrie CL , Holt KE . Unicycler: resolving bacterial genome assemblies from short and long sequencing reads. PLoS Comput Biol. 2017;13:e1005595. doi: 10.1371/journal.pcbi.1005595. 28594827 PMC5481147

[36] Kearse M , Moir R , Wilson A , Stones-Havas S , Cheung M , Sturrock S , Buxton S , Cooper A , Markowitz S , Duran C , et al. Geneious basic: an integrated and extendable desktop software platform for the organization and analysis of sequence data. Bioinformatics. 2012;28:1647–1649. doi: 10.1093/bioinformatics/bts199. 22543367 PMC3371832

[37] Hockenberry AJ , Wilke CO . BACPHLIP: predicting bacteriophage lifestyle from conserved protein domains. PeerJ. 2021;9:e11396. doi: 10.7717/peerj.11396. 33996289 PMC8106911

[38] Urbanowicz M , Guziński A , Szulc Ż . PhageAI: a new approach to predicting the lifestyle of bacteriophages using proteinBERT and convolutional neural networks. bioRxiv. 2025. doi: 10.1101/2025.09.02.673651.

[39] Garneau JR , Depardieu F , Fortier L-C , Bikard D , Monot M . PhageTerm: a tool for fast and accurate determination of phage termini and packaging mechanism using next-generation sequencing data. Sci Rep. 2017;7:8292. doi: 10.1038/s41598-017-07910-5. 28811656 PMC5557969

[40] Laslett D , Canback B . ARAGORN, a program to detect tRNA genes and tmRNA genes in nucleotide sequences. Nucleic Acids Res. 2004;32:11–16. doi: 10.1093/nar/gkh152. 14704338 PMC373265

[41] Moraru C , Varsani A , Kropinski AM . VIRIDIC—A novel tool to calculate the intergenomic similarities of prokaryote-infecting viruses. Viruses. 2020;12:1268. doi: 10.3390/v12111268. 33172115 PMC7694805

[42] Meier-Kolthoff JP , Göker M . VICTOR: genome-based phylogeny and classification of prokaryotic viruses. Bioinformatics. 2017;33:3396–3404. doi: 10.1093/bioinformatics/btx440. 29036289 PMC5860169

[43] Letunic I , Bork P . Interactive tree of life (iTOL) v5: an online tool for phylogenetic tree display and annotation. Nucleic Acids Res. 2021;49:W293–W296. doi: 10.1093/nar/gkab301. 33885785 PMC8265157

[44] Gilchrist CL , Chooi Y-H . Clinker & clustermap. Js: automatic generation of gene cluster comparison figures. Bioinformatics. 2021;37:2473–2475. doi: 10.1093/bioinformatics/btab007. 33459763

[45] Nishimura Y , Yoshida T , Kuronishi M , Uehara H , Ogata H , Goto S , Valencia A . ViPTree: the viral proteomic tree server. Bioinformatics. 2017;33:2379–2380. doi: 10.1093/bioinformatics/btx157. 28379287

[46] Bolduc B , Jang HB , Doulcier G , You Z , Roux S , Sullivan MB . vConTACT: an iVirus tool to classify double-stranded DNA viruses that infect archaea and bacteria. PeerJ. 2017;5:e3243. doi: 10.7717/peerj.3243. 28480138 PMC5419219

[47] Cook R , Brown N , Redgwell T , Rihtman B , Barnes M , Clokie M , Stekel DJ , Hobman J , Jones MA , Millard A . INfrastructure for a PHAge REference database: identification of large-scale biases in the current collection of cultured phage genomes. Phage. 2021;2:214–223. 36159887 10.1089/phage.2021.0007 PMC9041510

[48] Okamoto K , Mudd J , Mangan J , Huang W , Subbaiah T , Marmur J . Properties of the defective phage of bacillus subtilis. J Mol Biol. 1968;34:413–428. doi: 10.1016/0022-2836(68)90169-1. 4999722

[49] Katsura I . Mechanism of length determination in bacteriophage lambda tails. Adv Biophys. 1990;26:1–18. doi: 10.1016/0065-227X(90)90004-D. 2150582

[50] Jin M , Cai L , Lu L , Yu M , Zhang R . Combined metabolomic and genomic analyses reveal phage-specific and infection stage-specific alterations to marine roseobacter metabolism. ISME Commun. 2025;5:ycaf047. doi: 10.1093/ismeco/ycaf047. 40206216 PMC11981692

[51] Poubanne F , Darii E , Mariage A , Elisée E , Sirvain P , Hassan C , Rivollier J , Fossey-Jouenne A , Perret A , Méheust R , et al. Functional convergence in Z-DNA biosynthesis highlighted by the characterization of nucleotide metabolism enzymes in bacteriophages. Nucleic Acids Res. 2026;54:gkag079. doi: 10.1093/nar/gkag079. 41657244 PMC12884076

[52] Shockman G , Daneo-Moore L , Kariyama R , Massidda O . Bacterial walls, peptidoglycan hydrolases, autolysins, and autolysis. Microb Drug Resist. 1996;2:95–98. doi: 10.1089/mdr.1996.2.95. 9158729

[53] Bateman A , Rawlings ND . The CHAP domain: a large family of amidases including GSP amidase and peptidoglycan hydrolases. Trends Biochem Sci. 2003;28:234–237. doi: 10.1016/S0968-0004(03)00061-6. 12765834

[54] Fitzgerald CB , Shkoporov AN , Sutton TDS , Chaplin AV , Velayudhan V , Ross RP , Hill C . Comparative analysis of *Faecalibacterium prausnitzii* genomes shows a high level of genome plasticity and warrants separation into new species-level taxa. BMC Genomics. 2018;19:1–20. doi: 10.1186/s12864-018-5313-6. 30547746 PMC6295017

[55] Camargo AP , Baltoumas FA , Ndela EO , Fiamenghi MB , Merrill BD , Carter MM , Pinto Y , Chakraborty M , Andreeva A , Ghiotto G , et al. A genomic Atlas of the human gut virome elucidates genetic factors shaping host interactions. bioRxiv. 2025. doi: 10.1101/2025.11.01.686033.

[56] Nishijima S , Nagata N , Kiguchi Y , Kojima Y , Miyoshi-Akiyama T , Kimura M , Ohsugi M , Ueki K , Oka S , Mizokami M , et al. Extensive gut virome variation and its associations with host and environmental factors in a population-level cohort. Nat Commun. 2022;13:5252. doi: 10.1038/s41467-022-32832-w. 36068216 PMC9448778

[57] Tadmor AD , Mahmoudabadi G , Foley HB , Phillips R . Identification and spatio-temporal tracking of ubiquitous phage families in the human microbiome. Front Microb. 2023;1:1097124. doi: 10.3389/frmbi.2022.1097124. PMC12993540 41852816

[58] Reyes A , Marcelo P , Alves J , Durham AM , Gruber A . Use of profile hidden Markov models in viral discovery: current insights. Adv Genom Genetics. 2017;7:29–45. doi: 10.2147/AGG.S136574.

[59] Larsen N , Vogensen FK , van den Berg FWJ , Nielsen DS , Andreasen AS , Pedersen BK , Al-Soud WA , Sørensen SJ , Hansen LH , Jakobsen M , et al. Gut microbiota in human adults with type 2 diabetes differs from non-diabetic adults. PLoS One. 2010;5:e9085. doi: 10.1371/journal.pone.0009085. 20140211 PMC2816710

[60] Shen XJ , Rawls JF , Randall TA , Burcall L , Mpande C , Jenkins N , Jovov B , Abdo Z , Sandler RS , Keku TO . Molecular characterization of mucosal adherent bacteria and associations with colorectal adenomas. Gut Microbes. 2010;1:138–147. doi: 10.4161/gmic.1.3.12360. 20740058 PMC2927011

[61] Mushtaq N , Hussain S , Zhang S , Yuan L , Li H , Ullah S , Wang Y , Xu J. Molecular characterization of alterations in the intestinal microbiota of patients with grade 3 hypertension. Int J Mol Med. 2019;44:513–522. 31173179 10.3892/ijmm.2019.4235 PMC6605625

[62] Yan Q , Gu Y , Li X , Yang W , Jia L , Chen C , Han X , Huang Y , Zhao L , Fang Z , et al. Alterations of the gut microbiome in hypertension. Front Cell Infect Microbiol. 2017;7:381. doi: 10.3389/fcimb.2017.00381. 28884091 PMC5573791

[63] Su Q , Tun HM , Liu Q , Yeoh YK , Mak JWY , Chan FK , Ng SC . Gut microbiome signatures reflect different subtypes of irritable bowel syndrome. Gut Microbes. 2023;15:2157697. doi: 10.1080/19490976.2022.2157697. 36573834 PMC9809927

[64] Huang D , Xia R , Chen C , Liao J , Wang D , Alvarez PJ , Yu P . Adaptive strategies and ecological roles of phages in habitats under physicochemical stress. TIM. 2024;32:902–916. doi: 10.1016/j.tim.2024.02.002. 38433027

[65] Göller PC , Elsener T , Lorgé D , Radulovic N , Bernardi V , Naumann A , Amri N , Khatchatourova E , Coutinho FH , Loessner MJ , et al. Multi-species host range of staphylococcal phages isolated from wastewater. Nat Commun. 2021;12:6965. doi: 10.1038/s41467-021-27037-6. 34845206 PMC8629997

[66] Liu R , Li Z , Han G , Cun S , Yang M . Bacteriophage ecology in biological wastewater treatment systems. Appl Microbiol Biotechnol. 2021;105:5299–5307. doi: 10.1007/s00253-021-11414-8. 34181033

[67] Zhu Y , Shang J , Peng C , Sun Y . Phage family classification under caudoviricetes: a review of current tools using the latest ICTV classification framework. Front Microbiol. 2022;13:1032186. doi: 10.3389/fmicb.2022.1032186. 36590402 PMC9800612

[68] Fitzgerald CB , Shkoporov AN , Upadrasta A , Khokhlova EV , Ross RP , Hill C . Probing the “dark matter” of the human gut phageome: culture assisted metagenomics enables rapid discovery and host-linking for novel bacteriophages. Front Cell Infect Microbiol. 2021;11:616918. doi: 10.3389/fcimb.2021.616918. 33791236 PMC8005731

[69] Camarillo-Guerrero LF , Almeida A , Rangel-Pineros G , Finn RD , Lawley TD . Massive expansion of human gut bacteriophage diversity. Cell. 2021;184:1098–1109. e1099. doi: 10.1016/j.cell.2021.01.029. 33606979 PMC7895897

[70] Breitbart M , Salamon P , Andresen B , Mahaffy JM , Segall AM , Mead D , Azam F , Rohwer F . Genomic analysis of uncultured marine viral communities. Proc Natl Acad Sci. 2002;99:14250–14255. doi: 10.1073/pnas.202488399. 12384570 PMC137870

[71] Krishnamurthy SR , Wang D . Origins and challenges of viral dark matter. Virus Res. 2017;239:136–142. doi: 10.1016/j.virusres.2017.02.002. 28192164

[72] Gao R , Zhu Y , Kong C , Xia K , Li H , Zhang X , Liu Y , Zhong H , Yang R , Chen C , et al. Alterations, interactions, and diagnostic potential of gut bacteria and viruses in colorectal cancer. Front Cell Infect Microbiol. 2021;11:657867. doi: 10.3389/fcimb.2021.657867. 34307189 PMC8294192

[73] Huang Y , Lu W , Zeng M , Hu X , Su Z , Liu Y , Yuan J , Li L , Zhang X , Wang X . Mapping the early life gut microbiome in neonates with critical congenital heart disease: multiomics insights and implications for host metabolic and immunological health. Microbiome. 2022;10:245. doi: 10.1186/s40168-022-01437-2. 36581858 PMC9801562

[74] Wu J , Chai T , Zhang H , Huang Y , Perry SW , Li Y , Duan J , Tan X , Hu X , Liu Y , et al. Changes in gut viral and bacterial species correlate with altered 1, 2-diacylglyceride levels and structure in the prefrontal cortex in a depression-like non-human primate model. Transl Psychiatry. 2022;12:74. doi: 10.1038/s41398-022-01836-x. 35194021 PMC8863841

[75] Koskella B , Brockhurst MA . Bacteria–phage coevolution as a driver of ecological and evolutionary processes in microbial communities. FEMS Microbiol Rev. 2014;38:916–931. doi: 10.1111/1574-6976.12072. 24617569 PMC4257071

