# Supplementary material for: Isolation and characterization of a novel lytic bacteriophage for Faecalibacterium prausnitzii, a key member of the human gut microbiota
Source: Gut Microbes Rep. 2026 Jul 29;3(1):2708416. doi: 10.1080/29933935.2026.2708416 (PMC13432834; doi:10.1080/29933935.2026.2708416)
Supplement: Supplementary Material — docx [file KGMR_A_2708416_SM2384.docx]

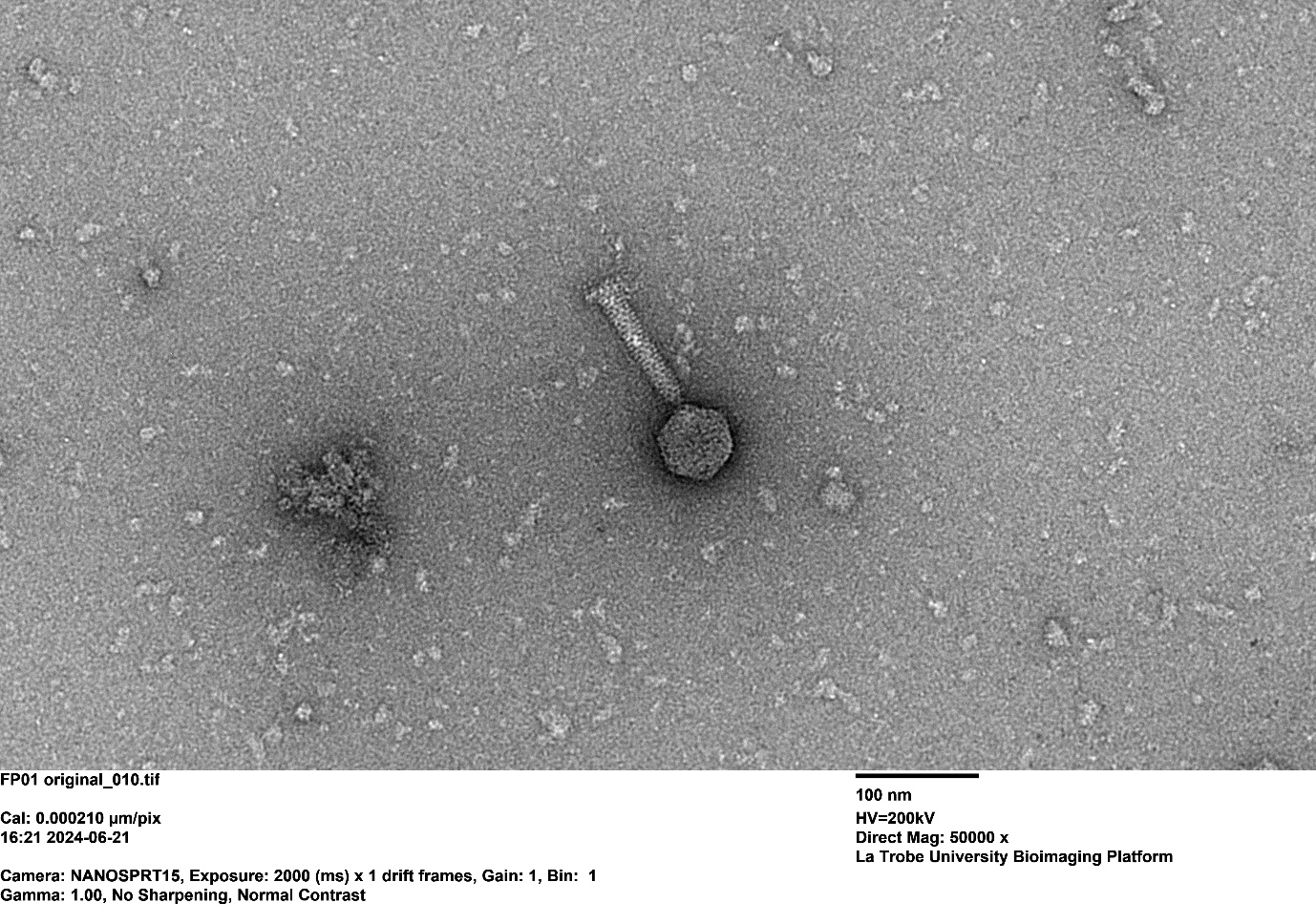


**Supplementary figure 1: Transmission electron micrograph of FP01.**


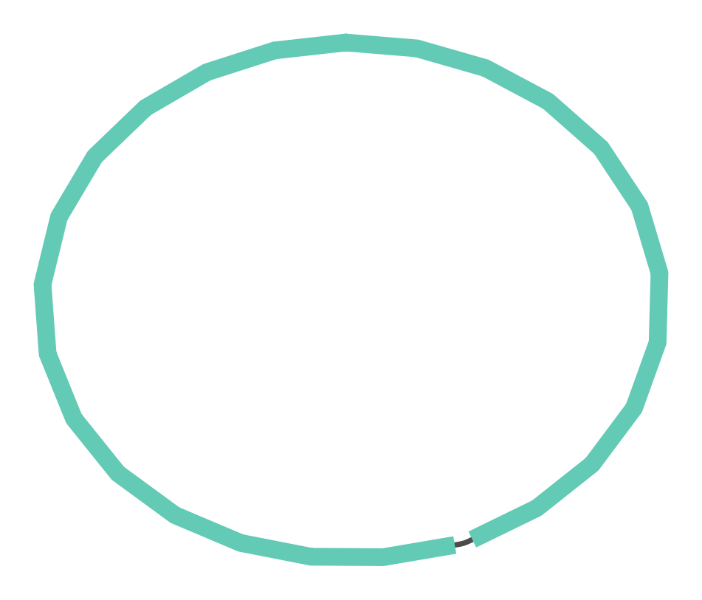


**Supplementary figure 2: Bandage assembly graph of FP01.** The FP01 genome was assembled into a single contig using Unicycler. Visualisation of a closed, loop structure with no unresolved repeats or branches, indicating a complete assembly.


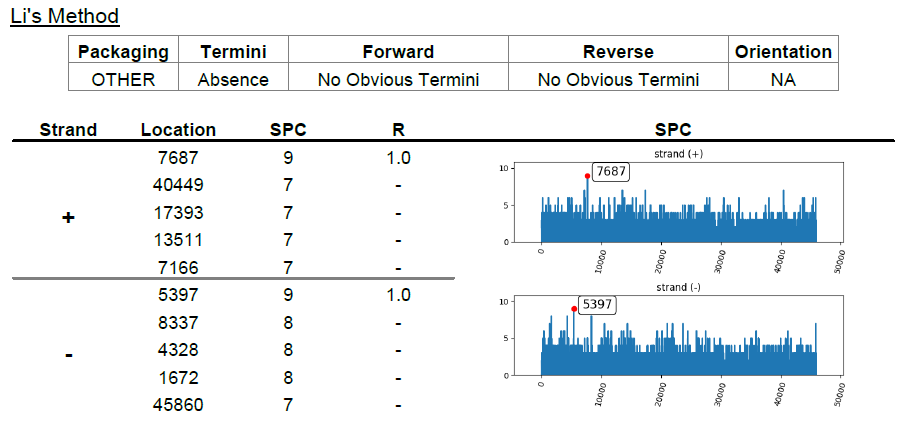


**Supplementary figure 3: Li’s method analysis of PhageTerm termini prediction.** The Li’s method dd not detect any obvious termini in FP01 and as so, classified the packaging category as OTHER.

**Supplementary table 1: Blastn comparison of FP01 genome to complete, whole genome *F. prausnitzii* sequences.** Assembly information, sample names, GenBank accession numbers, similarity to FP01 and presence of phage genes are indicated in the table below.

| Assembly | Samples | Accession | Similarity to FP01 | Presence of phage genes |
| --- | --- | --- | --- | --- |
| ASM2575776v1 | MAG: *F. prausnitzii* isolate KR001_HIC_0004 | CP107213.1 | 0 | Y |
| ASM5265979v1 | *F. prausnitzii* strain EB-FPDK11 | CP082235.1 | 0 | Y |
| ASM258694v1 | *F. prausnitzii* strain Indica | CP023819.1 | 0 | Y |
| ASM329363v1 | *F. prausnitzii* strain 942/30-2 | CP026548.1 | 0 | Y |
| ASM331246v1 | *F. prausnitzii* strain APC918/95b | CP030777.1 | 0 | Y |
| ASM1996795v1 | *F. prausnitzii* strain Fp944 | CP065382.1 | 0 | Y |
| ASM1996797v1 | *F. prausnitzii* strain Fp360 | CP065376.1 | 0 | No annotation |
| ASM1996799v1 | *F. prausnitzii* strain Fp77 | CP065377.1 | 0 | No annotation |
| ASM1996801v1 | *F. prausnitzii* strain Fp45 | CP065378.1 | 0 | No annotation |
| ASM1996803v1 | *F. prausnitzii* strain Fp40 | CP065379.1 | 0 | No annotation |
| ASM1996805v1 | *F. prausnitzii* strain Fp28 | CP065380.1 | 0 | No annotation |
| ASM1996807v1 | *F. prausnitzii* strain Fp1 | CP065381.1 | 0 | No annotation |
| ASM2874339v1 | *F. prausnitzii* strain AP34BHI | CP117963.1 | 0 | No annotation |
| ASM4002636v1 | *F. prausnitzii* strain 3 | CP157286.1 | 0 | Y |
| ASM4005898v1 | *F. prausnitzii* strain 4 | CP157368.1 | 0 | Y |
| ASM4005921v1 | *F. prausnitzii* strain 5 | CP157369.1 | 0 | Y |
| ASM4023458v1 | *F. prausnitzii* strain 6 | CP158110.1 | 0 | Y |
| ASM4794690v1 | *F. prausnitzii* strain 28 | CP181368.1 | 0 | Y |
| ASM4953293v1 | *F. prausnitzii* strain 5 | CP186690.1 | 0 | Y |
| ASM4953294v1 | *F. prausnitzii* strain 2 | CP186691.1 | 0 | Y |
| ASM4953295v1 | *F. prausnitzii* strain 1 | CP186692.1 | 0 | Y |
| Faecalibacterium_prausnitzii_TS_M3092 | *F. prausnitzii* strain TS M3092 | CP151836.1 | 0 | No annotation |
| ASM5120152v1 | *F. prausnitzii* strain UT1 | CP170812.1 | 0 | Y |
| UHGG_MGYG-HGUT-02545 | *F. prausnitzii* isolate MGYG-HGUT-02545 | LR699017.1 | 0 | No annotation |
| DSM32379 | *F. prausnitzii* isolate DSM32379 | OX636702.1 | 0 | No annotation |

**Supplementary table 2: Blast comparison of FP01 genome to Unified Human Gut Virome (UGHV) database.** A total of 71 matches were detected with the top ten outlined in the table below which includes their database ID, size, coordinates, number of matches (bp) and percent identity when compared to FP01.

| Match | Size (bp) | t_start | t_end | Matches (bp) | Percent identity |
| --- | --- | --- | --- | --- | --- |
| UHGV-1371713 | 45719 | 198 | 45674 | 29974 | 98.46 |
| UHGV-0202323 | 45965 | 201 | 45923 | 29425 | 98.32 |
| UHGV-1370750 | 45965 | 2262 | 45949 | 28468 | 98.35 |
| UHGV-1457728 | 45741 | 3683 | 45671 | 27528 | 98.29 |
| UHGV-0261444 | 45772 | 73 | 42031 | 27410 | 98.27 |
| UHGV-0390797 | 45722 | 8 | 41282 | 27132 | 98.35 |
| UHGV-1453813 | 46044 | 8890 | 46028 | 26936 | 98.82 |
| UHGV-1375229 | 45684 | 7 | 36523 | 26583 | 98.75 |
| UHGV-1448465 | 44826 | 1678 | 44816 | 26619 | 97.9 |
| UHGV-0828061 | 44717 | 8978 | 44706 | 25866 | 98.61 |

**Supplementary table 3: Species- and genus-level clustering of FP01 and related phage genome sequences based of VIRIDIC intergenomic similarity analysis.**

| genome | species_cluster | genus_cluster |
| --- | --- | --- |
| FP01 | 1 | 1 |
| Isolate_3552_106289 | 3 | 1 |
| Isolate_2423_15267 | 2 | 2 |
| Isolate_3584_27091 | 4 | 2 |

**Supplementary table 4: Pairwise intergenomic similarity (%) between FP01 and related viral genomes calculated by VIRIDIC.**

| genome | FP01 | Isolate_3552_106289 | Isolate_2423_15267 | Isolate_3584_27091 |
| --- | --- | --- | --- | --- |
| FP01 | 100 | 94.985 | 63.948 | 62.722 |
| Isolate_3552_106289 | 94.985 | 100 | 66.265 | 64.778 |
| Isolate_2423_15267 | 63.948 | 66.265 | 100 | 86.475 |
| Isolate_3584_27091 | 62.722 | 64.778 | 86.475 | 100 |
